# Physicochemical Characterization and Bibliometric Analysis of Malaysian Paracetamol Tablets in Compliance With MS ISO/IEC 17025 Standards

**DOI:** 10.1155/tswj/6619616

**Published:** 2026-09-08

**Authors:** Muhammad Hidhir Khawory, Norizamimie Nordin, Mohd Ferdaues Mohd Subki, Nur Izzati Abdul Ghani, Zalikha Zulkifli, Izyan Nur Wadhihah Azmy, Maisarah Mohamad Ridha, Azimah Amanah, Nurul Hanim Salin

**Affiliations:** ^1^ Malaysian Institute of Pharmaceuticals and Nutraceuticals, National Institutes of Biotechnology Malaysia, Gelugor, Pulau Pinang, Malaysia, ipharm-nibm.my

**Keywords:** bibliometric analysis, British Pharmacopoeia, Drug Registration Guidance Document, MS ISO/IEC 17025, paracetamol, physicochemical characterization, quality control

## Abstract

Ensuring the quality and safety of pharmaceutical products is essential for public health, particularly for widely used medications such as paracetamol. This study evaluated the physicochemical characteristics, dissolution performance, chromatographic quantification, heavy metal content, and microbiological quality of three paracetamol tablet brands, namely Dyna, Homecare Poro, and Paracil, under MS ISO/IEC 17025‐accredited laboratory conditions, complemented by a bibliometric analysis of global research trends in paracetamol quality control. Physicochemical assessments included uniformity of weight, hardness, friability, and disintegration time. Dissolution testing was performed using the ERWEKA paddle method at 37°C ± 0.5°C and 50 rpm to assess the rate and extent of drug release. The dissolution profiles showed variable release patterns among the tested brands, with Dyna achieving 100% drug release at 60 min, whereas Homecare Poro and Paracil showed lower release values at the same time point. Ultra high performance liquid chromatography (UHPLC) analysis showed distinct paracetamol peaks for all three brands, with Dyna producing the highest peak area response, followed by Homecare Poro and Paracil. Heavy metal analyses for lead (Pb), cadmium (Cd), mercury (Hg), and arsenic (As) were performed using flame atomic absorption spectrometry (FAAS) and flow injection atomic spectroscopy (FIAS). Microbiological tests included total aerobic microbial count (TAMC), total yeast and mold count (TYMC), bile‐tolerant gram‐negative bacteria, *Escherichia coli*, *Salmonella* spp., and *Staphylococcus aureus*. All tested parameters complied with British Pharmacopoeia (BP) and Drug Registration Guidance Document (DRGD) standards. Bibliometric analysis of 2191 Scopus‐indexed publications from 2010 to 2025 indicated strong research focus on dissolution, pharmaceutical analysis, and quality control, whereas heavy metal testing, microbiological evaluation, and accreditation‐based studies remain limited. This integrated analytical‐bibliometric approach confirms product compliance and highlights the need for robust accredited testing frameworks in pharmaceutical quality assurance.

## 1. Introduction

Paracetamol, or acetaminophen, is one of the most extensively consumed analgesic and antipyretic agents worldwide and is often combined with opioids, leading to more than 200 million prescriptions being dispensed annually [1]. Its broad therapeutic application and accessibility highlight the importance of rigorous quality assurance to safeguard public health [2]. Ensuring that paracetamol formulations consistently meet regulatory standards for identity, strength, purity, and performance is a fundamental requirement within the pharmaceutical industry [3]. Standard tablet quality assessments, such as uniformity of weight, hardness, friability, and disintegration time, are essential to confirm dosage accuracy, mechanical robustness, and appropriate drug release characteristics. In addition to these parameters, contamination with heavy metals, including lead (Pb), cadmium (Cd), arsenic (As), and mercury (Hg), represents a critical safety concern, as even trace‐level exposure may pose toxicological risks [4]. Compliance with established permissible limits is therefore necessary to prevent chronic or acute toxicity [5].

Microbiological evaluation is equally vital, as contamination by pathogenic or spoilage microorganisms can compromise product safety, stability, and therapeutic efficacy. Tests such as total aerobic microbial count (TAMC), yeast and mold enumeration, and screening for specific pathogens, including bile‐tolerant gram‐negative bacteria, *Escherichia coli*, *Salmonella* spp., and *Staphylococcus aureus*, are routinely required for nonsterile pharmaceuticals to ensure microbial safety throughout the product lifecycle [6]. Although several studies have assessed the physicochemical characteristics of paracetamol tablets, comparatively few have incorporated heavy metal testing or conducted analyses within MS ISO/IEC 17025‐accredited laboratories with an essential framework that ensures technical competence, validated analytical methodologies, and traceable measurement results accepted by regulatory bodies [7].

In parallel, bibliometric research examining global trends in paracetamol quality control remains limited, particularly in the context of heavy metal assessment, accreditation‐based testing, and emerging analytical priorities [8]. However, the recent Scopus‐based bibliometric analysis in this study reveals substantial and growing research output, with 2191 unique publications identified between 2010 and 2025 and notable increases in areas such as dissolution, pharmaceutical analysis, systematic reviews, and pharmacokinetics. These expanding research clusters suggest evolving scientific interest yet also highlight underexplored domains requiring further attention.

Accordingly, this study is aimed at (i) evaluating the physicochemical characteristics, heavy metal contents, and microbial safety of Dyna, Homecare Poro, and Paraciltablets under MS ISO/IEC 17025 accredited laboratory conditions and (ii) conducting a comprehensive bibliometric analysis to map global research trends in paracetamol quality control. Through this dual approach, the study seeks to identify current knowledge gaps, support evidence‐based regulatory practices, and guide future research directions in pharmaceutical quality assessment.

## 2. Materials and Methods

### 2.1. Sample Collection

Dyna, Homecare Poro, and Paracil paracetamol tablets were procured from a licensed pharmacy. The samples selected were within their shelf life and stored under the recommended conditions. The batch numbers for Dyna, Homecare Poro, and Paracil paracetamol tablets were 4924 (03), 0125 (05), and 2226 (04), respectively.

### 2.2. Laboratory Accuracy

All the analytical procedures were performed in an MS ISO/IEC 17025 accredited quality control laboratory following internally documented laboratory work instructions (LWIs) and quality assurance protocols.

### 2.3. Uniformity of Weight (Mass)

Twenty tablets were chosen at random. Each of the tablets was weighed using an analytical weighing balance. The average mass of 20 tablets was calculated and the percentage deviation of test specification according to average mass of tablets obtained was determined using the following expression: percentage deviation (*%*) = [(individual weight − average weight) / average weight] × 100. The specifications for tablets as stated in the British Pharmacopoeia (BP) [9], Volume V Appendix XII C (Ph. Eur. 2.9.5) are (i) ≤ 80 mg: ±10%, (ii) > 80 mg to < 250 mg: ±7.5%, and (iii) ≥250 mg: ±5%. The acceptance criteria for the tablets are that no more than two of the individual tablets may deviate from the average weight by the percentage listed, and no tablet may deviate by more than double that percentage.

### 2.4. Disintegration

Each of the six tubes in the basket was loaded with one dosage unit, and a disc was included when required. The test was carried out using the designated medium as the immersion fluid, maintained at a temperature of 37°C ± 2°C. After the set duration, the basket‐rack assembly was removed from the fluid, and the dosage units were examined. All units were found to have fully disintegrated. In cases where one or two units do not disintegrate, the procedure must be repeated with 12 additional units. The test is considered acceptable if at least 16 out of the 18 units tested have disintegrated ([9], Volume V Appendix XII A, [Ph. Eur. method 2.9.1]).

### 2.5. Hardness

Ten Tablets were chosen at random. Each of the tablets was placed between the jaws of hardness tester (ERWEKA TBH 125), taking into account the shape, break mark and inscription for each measurement orient of the tablet in the same way to the direction of the force application, where applicable. The measurement (thickness, diameter, and hardness) for 10 tablets was carried out. Any fragments from the previous tablets are cleared before each test. The average values of the forces measured were calculated using the following expression: Average forces measured of tablets (newton) = total forces measured of 10 tablets / number of tablets. The specification for tablets as stated in the BP [9] Volume V Appendix XVII H, (Ph. Eur. method 2.9.8), express the results as mean, minimum, and maximum values of the forces measured in newton. The acceptance criteria for the tablets are ±25.0% of standard deviation.

### 2.6. Friability

Ten tablets were chosen at random. Prior to analysis, the tablets were gently cleaned to remove any adhering dust. The sample was then precisely weighed (W_1_) and placed into the drum (ERWEKA TAR Series). The drum was rotated 100 times at a speed of 25 ± 1 revolutions per minute. After rotation, the tablets were taken out, any surface dust was removed as done previously, and the tablets were weighed (W_2_) again with accuracy. The percentage of mass loss (%) was determined by the following expression: Percentage of mass loss (*%*) = [(W_1_ − W_2_)/W_1_] × 100. The specification for tablets as stated in the BP [9], Volume V Appendix XVII G, (Ph. Eur. method 2.9.7) is a maximum loss of mass (obtained from a single test or from the mean of three tests) not greater than 1.0%. The acceptance criteria for tablets are the sample fails the test if obviously cracked, cleaved, or broken tablets are observed from the tablets after tumbling and the test shall be repeated twice if the weight loss is greater than the targeted value.

#### 2.6.1. Dissolution Testing

The dissolution study was conducted to determine the rate and extent of paracetamol release from three commercial tablet samples, namely Homecare Poro, Dyna, and Paracil. The test was performed using the ERWEKA dissolution apparatus under controlled conditions. A 0.1 M hydrochloric acid (HCl) medium was prepared by carefully adding 8.5 mL of concentrated HCl into approximately 800 mL of distilled water, followed by dilution to 1000 mL. The pH of the acidic medium was verified within the range of pH 1.0–1.2. Each dissolution vessel was filled with 900 mL of the prepared medium and maintained at 37°C ±0.5°C. The paddle method was used at a rotation speed of 50 rpm. One tablet was placed into each vessel, and the dissolution process was allowed to proceed under the specified conditions (BP Appendix XII B [Ph. Eur. method 2.9.3]).

Samples were withdrawn from the midpoint of each vessel at selected time intervals of 15, 30, 45, and 60 min. After each withdrawal, the sampled volume was replaced with fresh dissolution medium maintained at the same temperature to preserve sink condition and constant volume. The collected samples were diluted by transferring 500 *μ*L of sample into 49.5 mL of distilled water. The absorbance of each diluted sample was then measured using a UV‐visible spectrophotometer at 243 nm. A standard calibration curve with the equation *y* = 0.0872*x*–0.0003 and correlation coefficient *R* ^2^ = 0.9996 was used to determine the concentration of paracetamol in the samples. The amount of drug dissolved and the percentage of drug release were subsequently calculated based on the concentration obtained from the calibration curve (BP Appendix XII B [Ph. Eur. method 2.9.3]).

#### 2.6.2. Ultra High Performance Liquid Chromatography (UHPLC) Analysis

The analysis of paracetamol in the dissolution samples was performed using an Agilent 1290 Infinity LC system equipped with a diode array detector (DAD). Separation was carried out using an RRHD Eclipse Plus C18 column with dimensions of 2.1 × 50 mm and particle size of 1.8 *μ*m. Prior to analysis, the dissolution samples were prepared using a water:methanol mixture at a ratio of 70:30. For chromatographic separation, the mobile phase consisted of water and acetonitrile at a ratio of 50:50 under isocratic elution. The flow rate was maintained at 0.4 mL/min, with a total run time of 0.6 min for each injection. The injection volume was set at 4 *μ*L, and the analysis was performed at ambient temperature, approximately 24°C–25°C. Paracetamol was detected using DAD at a wavelength of 275 nm. The concentration of paracetamol in each sample was determined by comparing the peak response with the prepared standard calibration curve (In house method Agilent HPLC).

### 2.7. Limit Test of Heavy Metals

All limit tests for heavy metals were based on the in‐house method IP/LTM/008 by atomic absorption spectrometry with a Perkin Elmer microwave preparation system (MPS) Titan and American Public Health Association (APHA) 3111B, 3112B, and 3114C.

#### 2.7.1. Standard Sample Preparation Process

The test sample (0.5 g) was accurately weighed via an analytical balance (METTLER TOLEDO XS204), and the sample was transferred into a digestion vessel (Vessel 1). Slowly add 10.0 mL of concentrated nitric acid (HNO_3_), allowing fumes to release, and wait at least 10 min before sealing the vessel. The procedure was repeated in another vessel (Vessel 2) to ensure duplicate digestion.

#### 2.7.2. Microwave Digestion System

The samples were then subjected to microwave digestion via a Perkin Elmer Titan MPS system. This system helps to completely dissolve the samples for accurate analysis. The digestion vessels are subjected to high temperatures and pressures inside the microwave system, which enhances the breakdown of sample matrices.

#### 2.7.3. Postdigestion Preparation

After digestion, the test solutions are transferred into separate beakers for further processing. For Cd, 15.0 mL of the solution was transferred without requiring dilution. For As, 1.0 mL of the solution was transferred into a 100 mL beaker, mixed with 5.0 mL of HCl and 5.0 mL of 5% (potassium iodide [KI] with ascorbic acid), reduced for 45 min, and diluted to 50.0 mL. For Hg, 5.0 mL of the solution was transferred into a 100 mL beaker, mixed with 10 mL of 10% HCl, diluted to 50.0 mL with distilled water and treated with 1–2 drops of 5% potassium permanganate (KMnO_4_) solution. Pb requires the transfer of 19.0 mL of the solution without further dilution.

#### 2.7.4. Blank Sample Preparation

A blank sample was prepared by adding 10.0 mL of concentrated HNO_3_ into a separate beaker without any test material. This ensures that no contaminants are present in the reagents or glassware.

#### 2.7.5. Atomic Absorption Spectrometry Analysis

To set up the flame system for Cd and Pb analysis, the instruments flame atomic absorption spectrometry (FAAS) Perkin Elmer, computer, and fume ventilation are switched on, along with the gas supply. The “Syngistic for AA” software is opened, and the “Flame” technique is selected. The method is created or opened, and a hollow cathode lamp (HCL) for Cd and an electrodeless discharge lamp (EDL) for Pb are installed and automatically aligned. The HCL lamps warm for 15 min, and the EDL lamps warm for 45 min. The burner system is set up, safety checks are performed, and the flame is ignited. If necessary, the nebulizer is set up, followed by autobier head alignment and nebulizer gas flow optimization using 2 ppm Cu, ensuring that the absorbance is greater than 0.250 Abs.

For As and Hg analysis via the flow injection for atomic spectroscopy (FIAS) Perkin Elmer FIAS 100 system, the instruments, computer, and fume ventilation are switched on, along with the gas supply. The “Syngistic for AA” software is opened, and the “FIAS‐Flame” technique is selected. The method is created or opened, and the EDL lamps for As and Hg are manually installed and aligned, warming for 45 min. The burner system is set up, safety checks are performed, and the flame is ignited. The flow‐injection system is started, followed by T quartz cell alignment and burner alignment. The flow rate for the carrier was set between 9 and 11 mL/min, and the reductant was set between 5 and 7 mL/min.

The analysis was performed by preparing the test items, standards, reagents, blanks, carriers, and reductants. The Flame and FIAS systems are set up as described. A test item information file is created, and the analysis is carried out via manual sampling. Data are recorded before the system is shut down.

### 2.8. Microbial Limit Test (BP [9], Volume V Appendix XVI B [Ph. Eur. Chapters 2.6.12 and 2.6.13])

#### 2.8.1. Sampling

First, 10 g (solid form)/10 mL (liquid form) and 25 g (solid form)/25 mL (liquid form) of the samples were weighed on a weighing balance before being transferred into two different sterile containers, ensuring that the containers were sealed completely after the sampling process.

#### 2.8.2. Sample Preparation

For microbiological testing involving TAMC, TYMC, *E. coli*, bile‐tolerant gram‐negative bacteria, *S. aureus* and *Salmonella* spp., aseptic conditions were maintained throughout the procedure. Solid and liquid samples were prepared by transferring 10 g or 10 mL, respectively, into 90 mL of soybean casein digest broth (SCDB). The mixture was gently agitated until the sample was fully dissolved. A 1:10 serial dilution was subsequently prepared using SCDB, and this suspension was used for the enumeration of colony‐forming units (CFU/mL). For samples identified as nonfatty or poorly soluble in water, SCDB supplemented with 1 g/L polysorbate 80 was utilized to enhance dispersion. The pH of the mixture was adjusted to within the range of 6.0–8.0, if necessary, to ensure optimal microbial recovery conditions. For the detection of *Salmonella* spp., sample preparation was carried out using a larger inoculum volume. A total of 25 g (solid) or 25 mL (liquid) of the sample was transferred into 225 mL of SCDB, followed by gentle mixing until complete dissolution was achieved. This enrichment broth was then incubated for further selective testing.

#### 2.8.3. Microbial Morphology Analysis

##### 2.8.3.1. TAMC and TYMC

After the incubation period, the plates were examined for microbial growth. Colonies observed on soybean casein digest agar (SCDA) for TAMC and on Sabouraud dextrose agar (SDA) for TYMC were counted. The counts obtained from both plates were averaged, and the final value was expressed based on the corresponding dilution factor of the sample.

##### 2.8.3.2. Test for Specific Microorganisms

The morphology of the growth (if present) on the inoculated plates was observed. For identification of the microorganisms, refer to Table 1 below for the morphology of the bacteria on the plates.

**Table 1:** Morphology of the tested microorganisms.

Test organism	Growth characteristics
*Escherichia coli*	Creamy beige colonies
*Staphylococcus aureus*	Circular, smooth, low convex, and yellow pigmented
*Salmonella* spp.	Red colonies, some with black centers

##### 2.8.3.3. Test for Bile‐Tolerant Gram‐Negative Bacteria

The appearance of microbial colonies was interpreted as a positive result. The minimum sample quantity producing a positive outcome and the maximum quantity yielding a negative outcome were recorded accordingly. The corresponding probable bacterial count is presented in Table 2.

**Table 2:** Summary of result interpretation criteria.

Microbiological results by sample quantity			Probable number of bacteria per g or mL of sample
0.1 g or	0.01 g or	0.001 g or	
0.1 mL	0.01 mL	0.001 mL	
+	+	+	Greater than 10^3^
+	+	—	Less than 10^3^ and greater than 10^2^
+	—	—	Less than 10^2^ and greater than 10
—	—	—	Less than 10

#### 2.8.4. Bibliometric Analysis

The study was conducted exclusively with 2191 data retrieved from the Scopus database, owing to its comprehensive coverage of peer‐reviewed literature in the pharmaceutical and analytical sciences. The objective was to explore publication trends, citation impacts, and research patterns related to paracetamol tablet analysis, physicochemical evaluation, and quality control under the framework of MS ISO/IEC 17025 compliance. The search strategy was designed with Boolean operators and field‐restricted terms to ensure precision and relevance. The complete search query used for data retrieval is presented in (Table 3).

**Table 3:** Search strings in Scopus databases.

Database	Search string
Scopus	TITLE‐ABS KEY ((“Paracetamol” OR “Acetaminophen”) AND (“Physico‐chemical” OR “Tablet characterization” OR “Quality control” OR “Dissolution” OR “Pharmaceutical analysis” OR “heavy metal” OR “ISO 17025” OR “accreditation” OR “microbial limit test” OR “TAMC” OR “TYMC” OR “E.coli” OR “Salmonella”)) AND PUBYEAR >2009 AND PUBYEAR <2027

The search was performed in September 2025, and the retrieved dataset was exported in CSV formats containing bibliographic metadata such as author names, publication titles, journal sources, keywords, abstracts, citation counts, and country affiliations. Data preprocessing was carried out to ensure analytical accuracy, involving the removal of duplicate records, exclusion of nonscientific items such as conference abstracts or short notes, and normalization of author and institution names to unify variations in spelling and formatting. The refined dataset represented a high‐quality corpus suitable for statistical and network‐based analyses.

The bibliometric indicators assessed included annual publication growth, citation performance, the most productive authors and institutions, leading journals, and keyword co‐occurrence networks. Analytical and visualization tasks were performed via the ScientoPy package [10], a Python‐based tool specialized for bibliometric data mining and trend analysis. ScientoPy enabled the generation of temporal publication graphs, collaboration maps, and keyword clustering networks, which collectively provided insights into evolving research interests, interdisciplinary linkages, and emerging themes in paracetamol formulation and analytical standardization. The results from this bibliometric workflow serve as a foundation for understanding the global research landscape on paracetamol tablets, particularly within the context of analytical quality assurance and MS ISO/IEC 17025 laboratory compliance.

## 3. Results and Discussion

As shown in Table 4, Dyna, Homecare Poro, and Paracil paracetamol tablets comply with the pharmacopeial standards. According to BP [9], the acceptable variation in weight (mass) for general uncoated tablets is within ±5.0% (Appendix XII C, Ph. Eur. method 2.9.5), and the disintegration time must not exceed 30 min (Appendix XII A, Ph. Eur. method 2.9.1). The friability limit for such tablets is set at not more than 1.0% (Appendix XVII G, Ph. Eur. method 2.9.7), whereas the acceptable range for hardness variation is ±25.0% (Appendix XVII H, Ph. Eur. method 2.9.8) [11]. As for elemental impurities, the uncoated tablets also conform to the heavy metals limit test outlined in the Drug Registration Guidance Document (DRGD), 2025, as summarized in Table 5. The maximum allowable concentration is 10.0 mg/kg or mg/L (10.0 ppm) for Pb, 0.3 mg/kg or mg/L (0.3 ppm) for Cd, 0.5 mg/kg or mg/L (0.5 ppm) for Hg, and 5.0 mg/kg or mg/L (5.0 ppm) for As. The standard calibration curve showed good linearity with an *R* ^2^ value of 0.9996, indicating that the UV‐visible spectrophotometric method was suitable for estimating paracetamol concentration within the tested range. The evaluation of Pb, Cd, Hg, and As is important because these elements may enter pharmaceutical products through contaminated raw materials, excipients, water used during manufacturing, processing equipment, environmental exposure, or packaging materials. In tablet formulation, heavy metal contamination may also occur from mineral‐based excipients, catalysts, manufacturing residues, or inadequate control of the production environment.

**Table 4:** Parameters and results in accordance with BP.

Parameter	Result			Specification	Status
	Dyna	Homecare Poro	Paracil		
Uniformity of weight (mass)	554.12^a^ ± 7.38 mg	604.65^a^ ± 3.09 mg	566.08^a^ ± 8.17 mg	5%	Comply
Hardness	221.92^a^ ± 32.5 N	177.78^a^ ± 9.97 N	147.42^a^ ± 26.0 N	25%	Comply
Friability	0.15%^a^ ±0.04%	0.38%^a^ ± 0.01%	0.18%^a^ ± 0.02%	NMT 1%	Comply
Disintegration time	8:39^a^ ± 1:33	3:04^a^ ± 0:10	1:30^a^ ± 0:09	NMT 30 min	Comply
Total aerobic microbial count (TAMC)	0^b^	0^b^	0^b^	NMT 2 × 10^3^ CFU/g or CFU/mL	Comply
Total yeast and mold count (TYMC)	0^b^	0^b^	0^b^	NMT 2 × 10^2^ CFU/g or CFU/mL	Comply
Bile‐tolerant gram‐negative bacteria	0^c^	0^c^	0^c^	NMT 1 × 10^2^ CFU/g or CFU/mL	Comply
*Escherichia coli*	Absent	Absent	Absent	Absent in 1 g or 1 mL	Comply
*Salmonella*	Absent	Absent	Absent	Absent in 25 g or 25 mL	Comply
*Staphylococcus aureus*	Absent	Absent	Absent	Absent in 1 g or 1 mL	Comply

^a^Values represent the mean of five determinations ± standard deviation.

^b^Values represent the mean of two determinations.

^c^Values represent the mean of three determinations.

**Table 5:** Parameters and results in accordance with the Drug Registration Guidance Document (DRGD).

Parameter	Result			Specification	Status
	Dyna	Homecare Poro	Paracil		
Cadmium (Cd)	−1.212 ± 0.2038 mg/kg	−0.600 ± 0.0301 mg/kg	0.297 ± 0.0214 mg/kg	NMT 0.3 mg/kg or 0.3 mg/L	Comply
Plumbum (Pb)	−6.106 ± 0.3112 mg/kg	−19.22 ± 6.903 mg/kg	−8.218 ± 0.4775 mg/kg	NMT 10.0 mg/kg or 10.0 mg/L	Comply
Arsenic (As)	0.005 ± 0.0036 mg/kg	−0.001 ± 0.0047 mg/kg	−0.012 ± 0.0017 mg/kg	NMT 5.0 mg/kg or 5.0 mg/L	Comply
Mercury (Hg)	−0.005 ± 0.0051 mg/kg	0.041 ± 0.0033 mg/kg	0.000 ± 0.0043 mg/kg	NMT 0.5 mg/kg or 0.5 mg/L	Comply

*Note:* Each value is the average of three analyses ± standard deviation.

The dissolution results showed that the percentage of drug release varied among the three tablet samples Table 6. For Homecare Poro, the percentage release was 78.6% at 15 min, increased slightly to 79.2% at 30 min, then decreased to 67.7% at 45 min and further dropped to 25.0% at 60 min. Dyna demonstrated the highest initial release, with 90.6% drug release at 15 min. However, the release decreased to 66.7% at 30 min and 69.8% at 45 min before reaching 100.0% at 60 min. Paracil showed 84.4% release at 15 min, followed by a decrease to 66.7% at 30 min, 66.1% at 45 min, and 32.8% at 60 min. Overall, the dissolution profiles of all three brands did not show a consistent cumulative release pattern. In a typical dissolution study, the percentage of drug released is expected to increase progressively over time as more drug dissolves into the medium. However, the observed results showed fluctuating or decreasing values, particularly for Homecare Poro and Paracil. Dyna achieved complete release at 60 min but still showed an irregular trend at earlier time points.

**Table 6:** Dissolution of Homecare Poro, Dyna, and Paracil tablets.

Dissolution of paracetamol 500 mg												
Sample	Concentration (*μ*g/mL)				Amount dissolved (mg)				% Drug release (%)			
	15 min	30 min	45 min	60 min	15 min	30 min	45 min	60 min	15 min	30 min	45 min	60 min
Homecare Poro	0.0151	0.0152	0.013	0.0048	15.1	15.2	13	4.8	78.6	79.2	67.7	25
Dyna	0.0174	0.0128	0.0134	0.0192	17.4	12.8	13.4	19.2	90.6	66.7	69.8	100
Paracil	0.0162	0.0128	0.0127	0.0063	16.2	12.8	12.7	6.3	84.4	66.7	66.1	32.8

**Figure 1:**
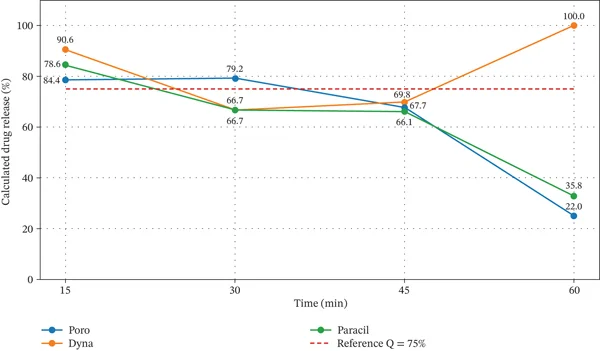
Comparative calculated drug‐release profiles of Homecare Poro, Dyna, and Paracil paracetamol tablets at 15, 30, 45, and 60 min. The dashed line represents the general reference value of *Q* = 75*%*.

Figure 1 compares the calculated drug‐release profiles of Homecare Poro, Dyna, and Paracil tablets over 60 min. At 15 min, the calculated release values were 78.6%, 90.6%, and 84.4% for Homecare Poro, Dyna, and Paracil, respectively. Homecare Poro maintained a value above the general *Q* = 75*%* reference at 30 min, whereas Dyna and Paracil recorded 66.7%. At 45 min, the calculated release values for all three brands were below 75%. At 60 min, Dyna reached 100.0%, whereas Homecare Poro and Paracil recorded 25.0% and 32.8%, respectively. The differences may reflect variation in formulation, tablet hardness, disintegration behavior, excipient composition, sampling, dilution, or analytical procedures. The decline observed at later sampling times for Homecare Poro and Paracil is not characteristic of a conventional cumulative dissolution profile and indicates that the calculations and sampling corrections should be verified. Consequently, these results are interpreted as comparative calculated drug‐release profiles, and no definitive pharmacopoeia pass‐or‐fail conclusion is made.

**Figure 2:**
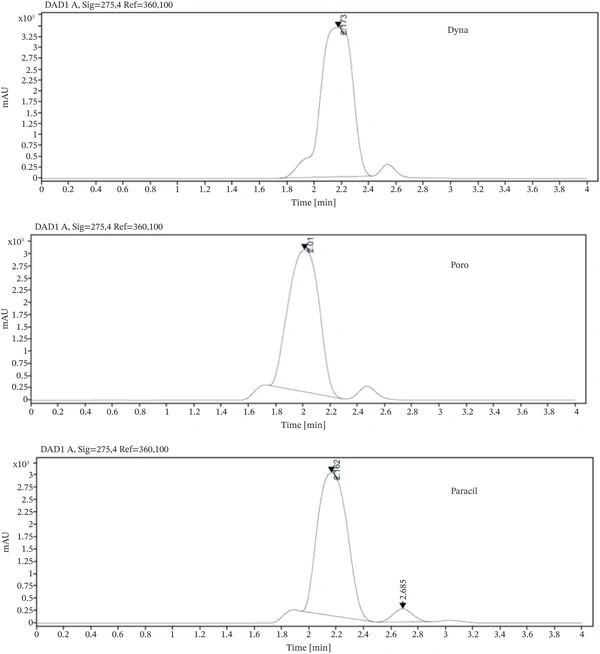
HPLC chromatographic profiles of paracetamol in Dyna, Homecare Poro, and Paracil tablet samples.

The UHPLC analysis of paracetamol was performed using detection at 275 nm, and the standard calibration curve showed excellent linearity with the regression equation *y* = 861.18*x*–0.3346 and an *R* ^2^ value of 1.0000. This indicates a strong linear relationship between paracetamol concentration and peak area within the tested calibration range. The chromatographic analysis of the tablet samples showed clear paracetamol peaks with retention times between approximately 2.17 min Figure 2. Homecare Poro produced a major peak at 2.010 min with a peak area of 45,742.62 mAU. Dyna produced a major peak at 2.173 min with the highest peak area of 56,581.15 mAU. Paracil produced a major peak at 2.162 min with a peak area of 43,740.46 mAU and an additional smaller peak at 2.685 min with a peak area of 2,715.89 mAU. Based on the calibration curve, Dyna showed the highest estimated paracetamol concentration, followed by Homecare Poro and Paracil. The calculated concentration Table 7 for Dyna was approximately 65.70 mg/mL, whereas Homecare Poro and Paracil showed estimated concentrations of approximately 53.12 and 50.79 mg/mL, respectively, when calculated using the main paracetamol peak area. If the total peak area of Paracil is considered, including the smaller secondary peak, the calculated value would be higher; however, the secondary peak should not be directly assigned to paracetamol unless confirmed by retention time matching or peak purity analysis. Therefore, the main peak was considered more appropriate for estimating paracetamol content.

**Table 7:** UHPLC quantification of paracetamol in tablet samples based on peak area response.

Sample	Labeled strength (mg/tablet)	Concentration (mg/mL)	Peak area (mAU)
Blank	0	0	0
Dyna	500	65.70	56581.15
Homecare Poro	500	53.12	45742.62
Paracil	500	50.79	43740.46

The BP outlines specific microbial limits for nonsterile pharmaceutical products. For oral preparations that are nonaqueous, the TAMC must not exceed 2 × 10^3^ CFU per gram or milliliter. In the case of aqueous oral preparations, the limit is set at 2 × 10^2^ CFU/g or mL. For total yeast and mold count (TYMC), nonaqueous formulations should remain within 2 × 10^2^ CFU/g or mL, whereas aqueous products are restricted to 2 × 10^1^ CFU/g or mL. Additionally, bile‐tolerant gram‐negative bacteria should not surpass 1 × 10^2^ CFU/g or mL across most nonsterile dosage forms. These microbial limits are based on the procedures and incubation parameters outlined in BP [9], Appendix XVI B (Ph. Eur. 2.6.12 and 2.6.13), with details provided in Table 4.

In nonsterile pharmaceutical dosage forms, *E. coli*, *Salmonella* spp., and *S. aureus* must not be present within specified sample quantities. In particular, *E. coli* must be absent in 1 g or 1 mL, *Salmonella* spp. must not be detected in 25 g or 25 mL, and *S. aureus* should be absent in 1 g or 1 mL of product. According to the microbial morphology evaluation (Tables 8, 9, and 10), no microbial colonies were detected for TAMC, TYMC, or bile‐tolerant gram‐negative organisms. Furthermore, *E. coli, Salmonella* spp., and *S. aureus* were not present in the tested paracetamol formulation. Meeting these criteria verifies that the product adheres to acceptable microbiological safety standards for patient use. Routine environmental monitoring provides documented evidence of the effectiveness of contamination‐control measures and helps ensure that microbial risks remain low [12]. This microbial evaluation is important because contamination may compromise product safety, stability, and patient health, particularly if pathogenic organisms are present. Possible sources of microbial contamination include raw materials, excipients, water used during manufacturing, equipment surfaces, personnel handling, environmental exposure, and improper storage conditions. For nonsterile medicines, microbiological quality testing is crucial for detecting excessive microbial loads or pathogenic organisms in raw materials as well as finished products. As these products may contain a limited microbial bioburden, risk assessment is therefore based on the type of formulation, intended purpose, and route of administration [13].

**Table 8:** Microbial morphology analysis for Dyna.

Test	Observations		
Total aerobic microbial count (TAMC)	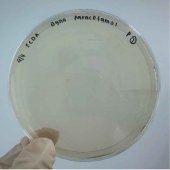 Plate 1: no microbial growth observed	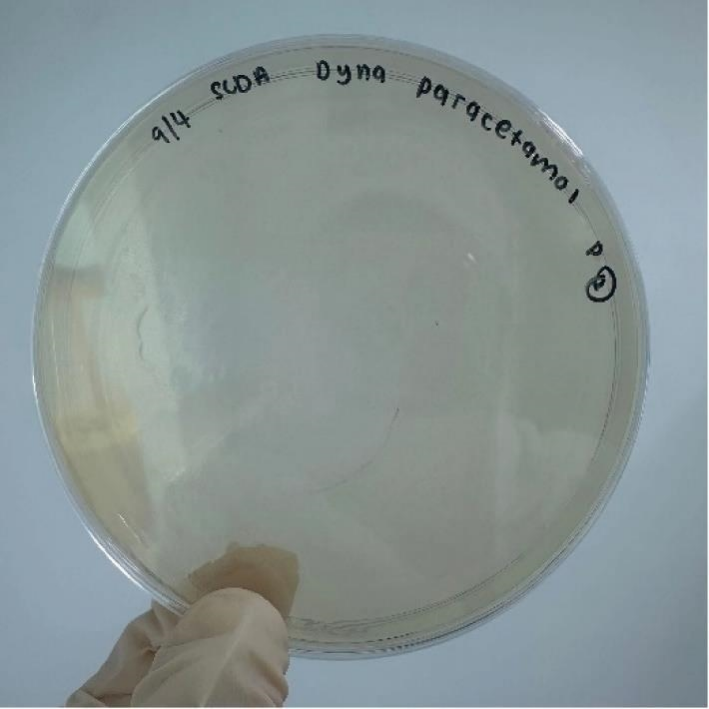 Plate 2: no microbial growth observed	
Total yeast and mold count (TYMC)	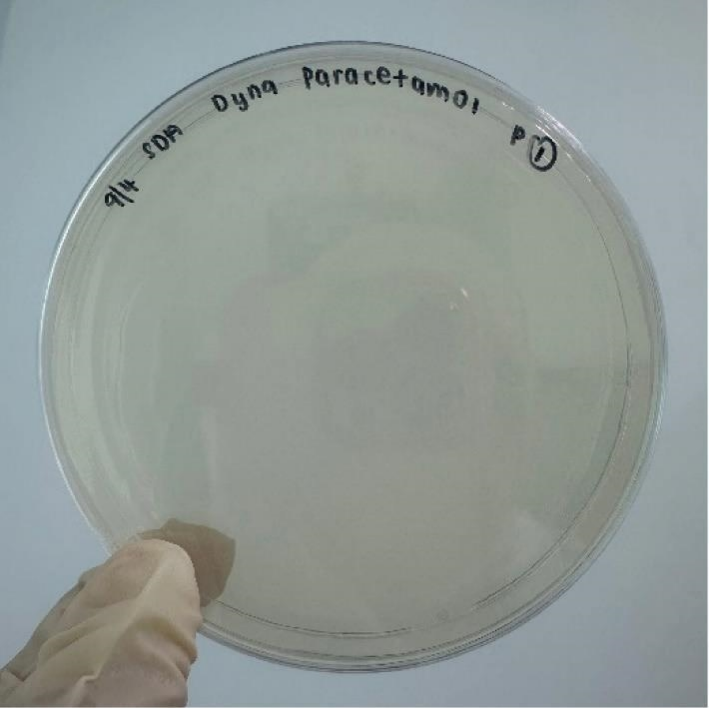 Plate 1: no microbial growth observed	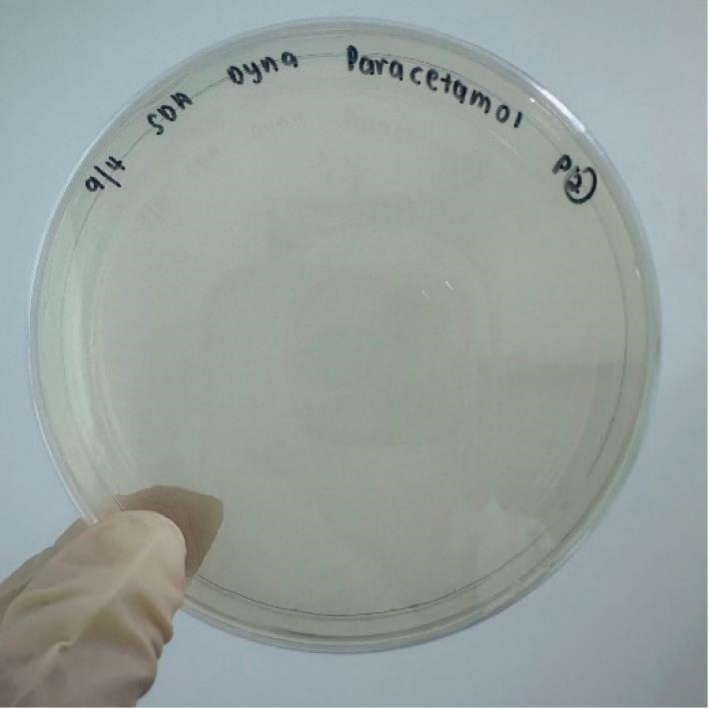 Plate 2: no microbial growth observed	
Bile‐tolerant gram‐negative bacteria	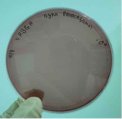 Plate 10^0^: no microbial growth observed	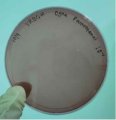 Plate 10^−1^: no microbial growth observed	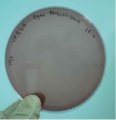 Plate 10^−2^: no microbial growth observed
*Escherichia coli*	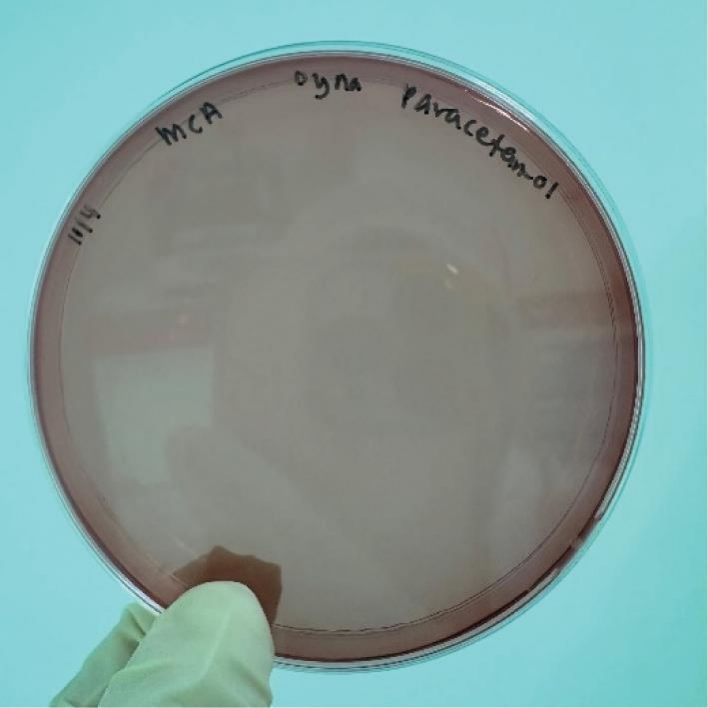 Absent		
*Salmonella* spp.	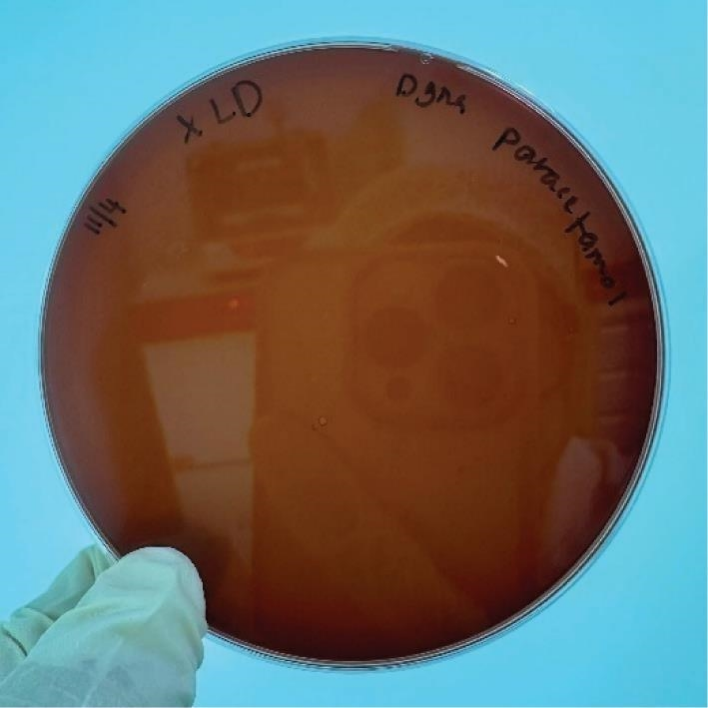 Absent		
*Staphylococcus aureus*	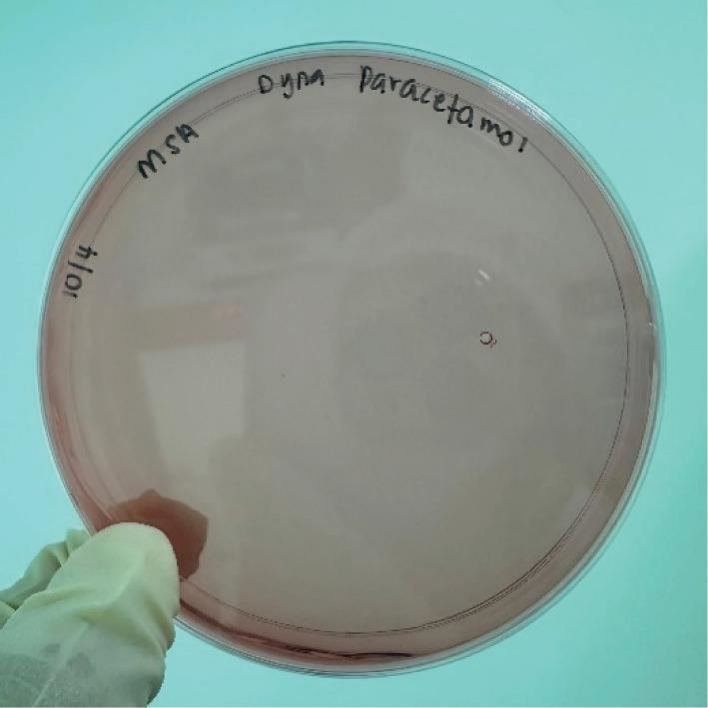 Absent		

**Table 9:** Microbial morphology analysis for Homecare Poro.

Test	Observations		
Total aerobic microbial count (TAMC)	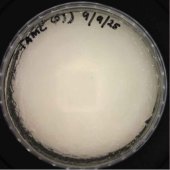 Plate 1: no microbial growth observed	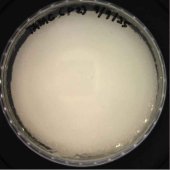 Plate 2: no microbial growth observed	
Total yeast and mold count (TYMC)	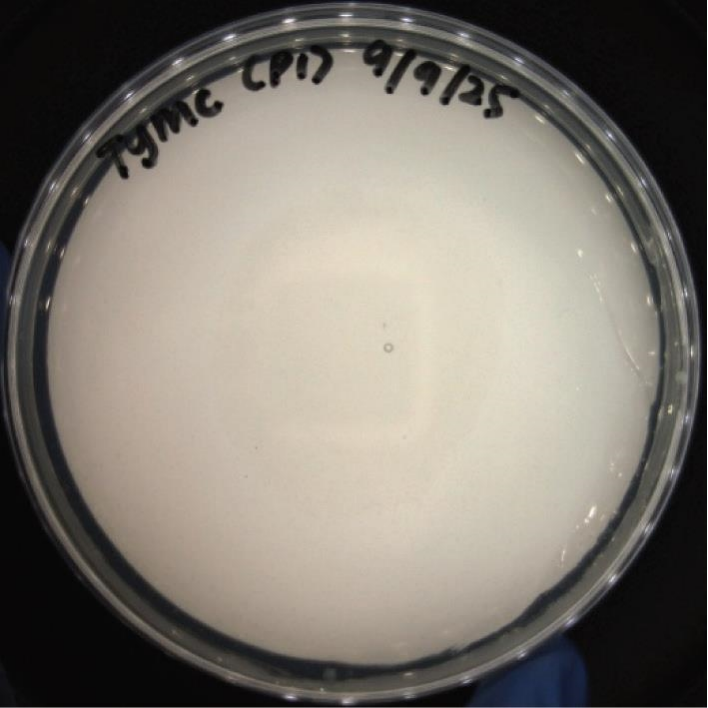 Plate 1: no microbial growth observed	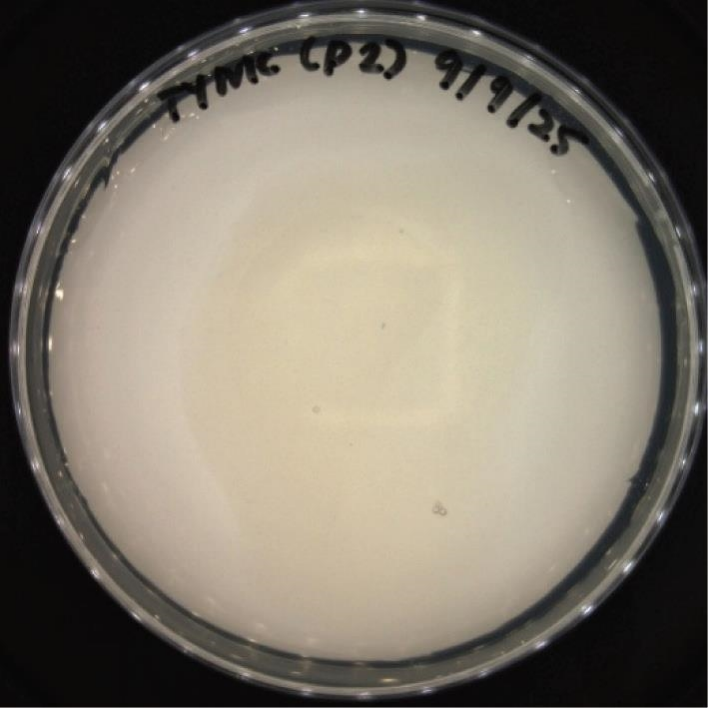 Plate 2: no microbial growth observed	
Bile‐tolerant gram‐negative bacteria	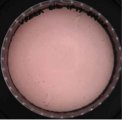 Plate 10^0^: no microbial growth observed	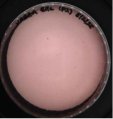 Plate 10^−1^: no microbial growth observed	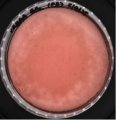 Plate 10^−2^: no microbial growth observed
*Escherichia coli*	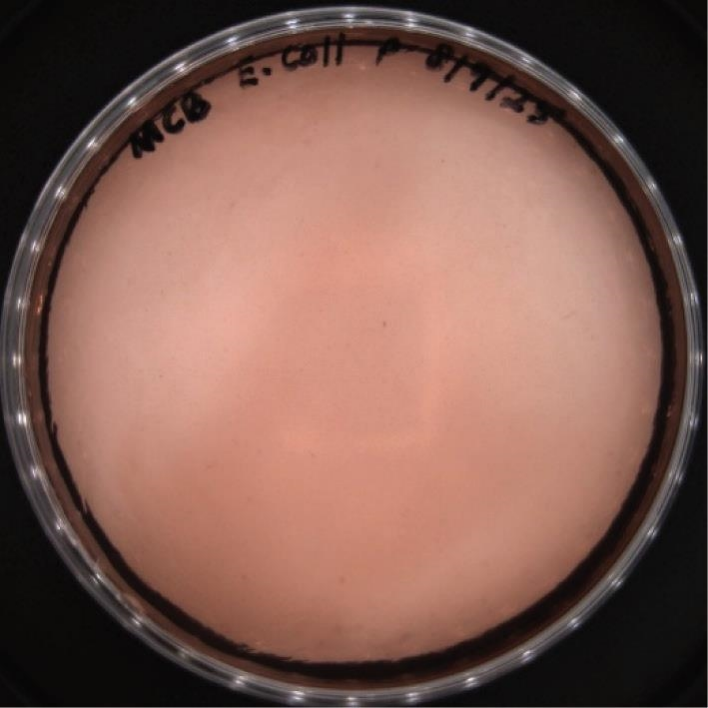 Absent		
*Salmonella* spp.	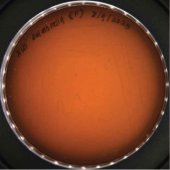 Absent		
*Staphylococcus aureus*	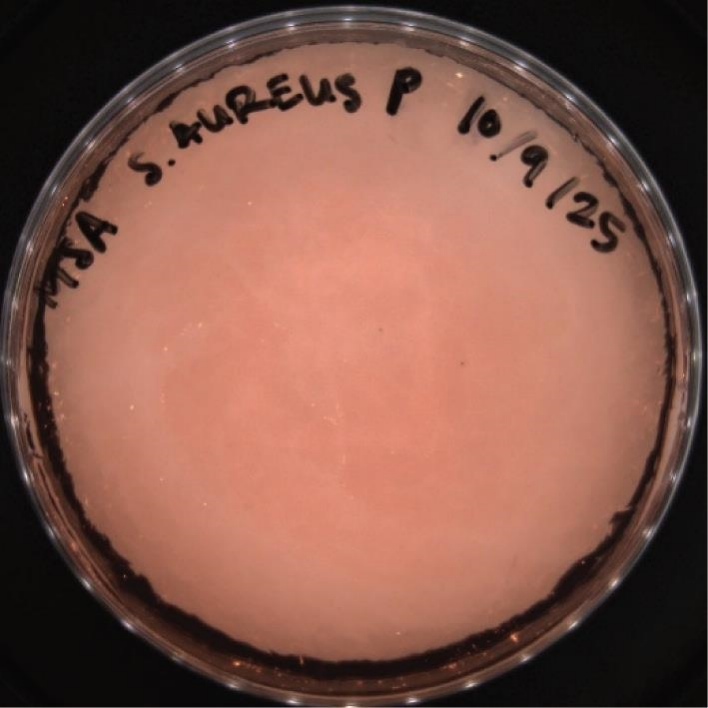 Absent		

**Table 10:** Microbial morphology analysis for Paracil.

Test	Observations		
Total aerobic microbial count (TAMC)	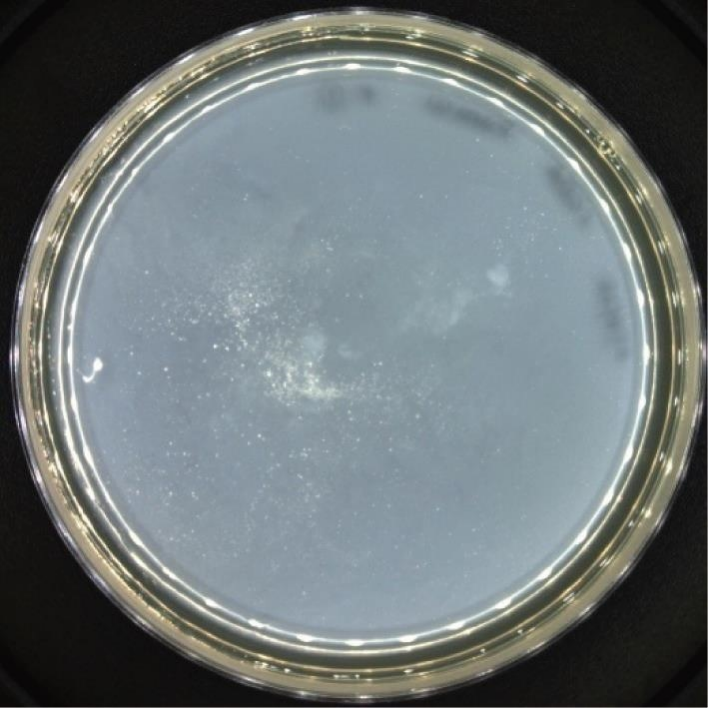 Plate 1: no microbial growth observed	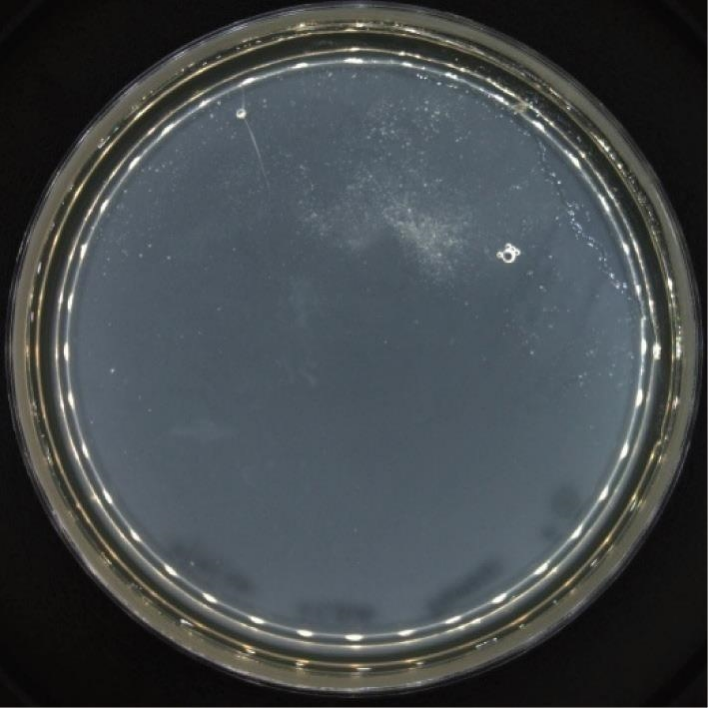 Plate 2: No microbial growth observed	
Total yeast and mold count (TYMC)	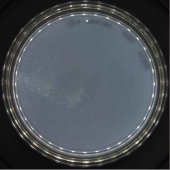 Plate 1: no microbial growth observed	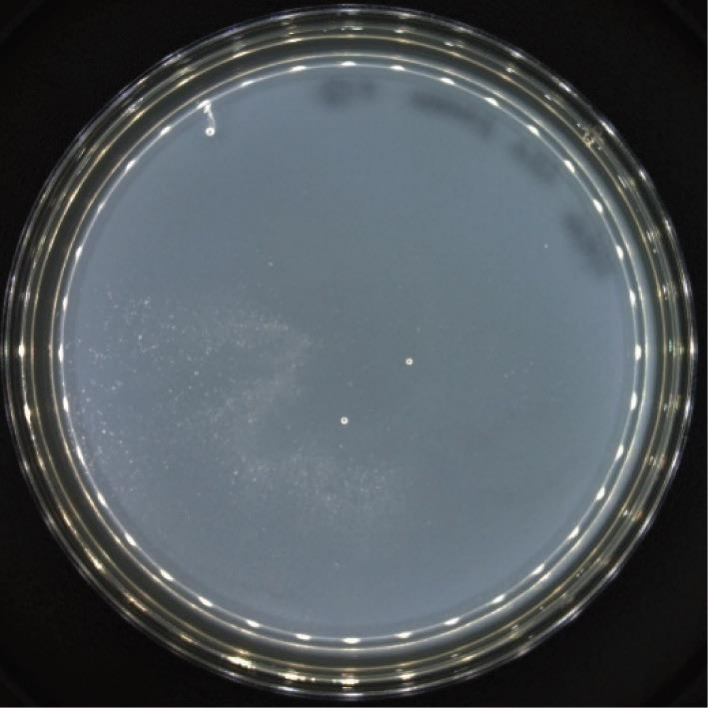 Plate 2: no microbial growth observed	
Bile‐tolerant gram‐negative bacteria	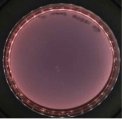 Plate 10^0^: no microbial growth observed	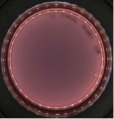 Plate 10^−1^: no microbial growth observed	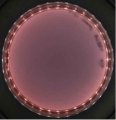 Plate 10^−2^: no microbial growth observed
*Escherichia coli*	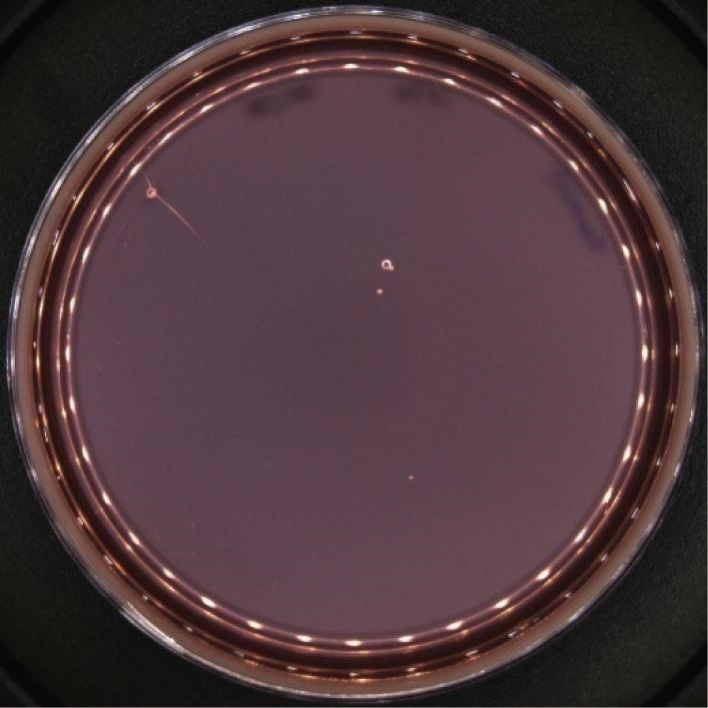 Absent		
*Salmonella* spp.	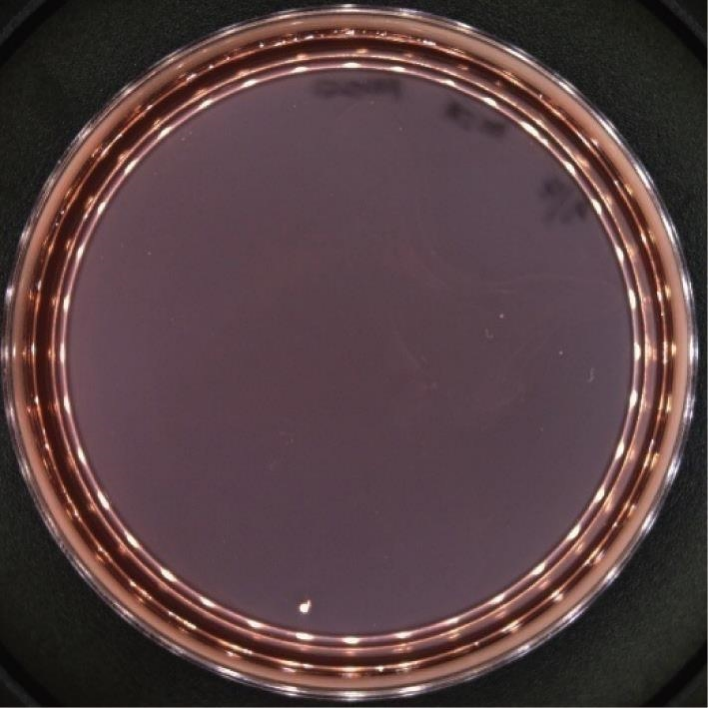 Absent		
*Staphylococcus aureus*	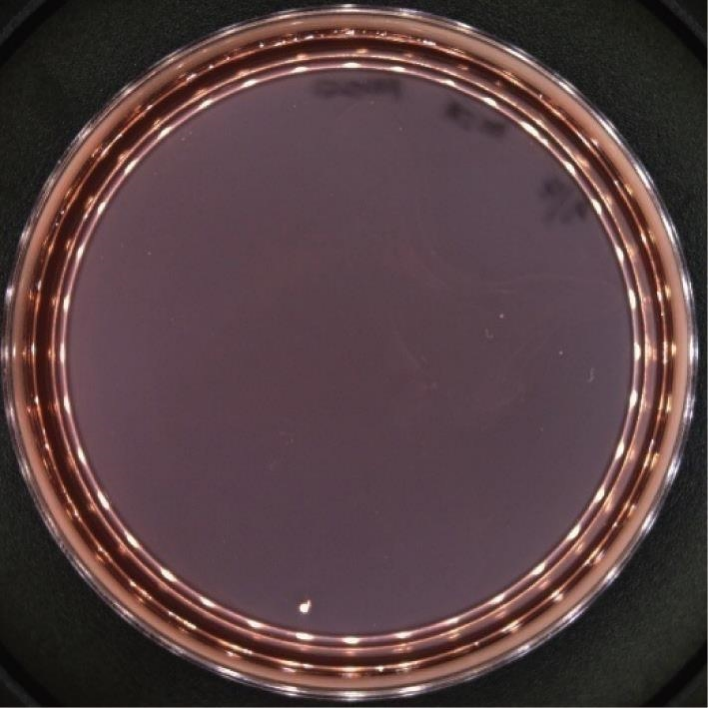 Absent		

**Figure 3:**
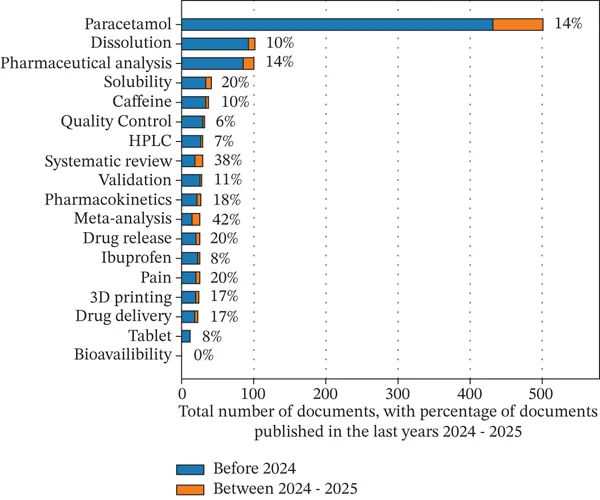
Author keyword trends.

The bibliometric analysis provides an informative overview of global research activity related to paracetamol quality control over the past two decades and reveals several important trends that complement the laboratory findings of this study. With 2191 unique publications retrieved from Scopus after deduplication, the dataset demonstrates a substantial and sustained research interest in paracetamol, driven largely by its widespread clinical use, safety considerations, and continuing relevance in pharmaceutical analysis. This bibliometric analysis was strengthened through quantitative and statistical evaluation assessed publication growth, citation impact, keyword frequency, co‐occurrence patterns, and thematic distribution to identify major research directions in paracetamol tablet quality assessment. Keyword frequency analysis (Figure 3) highlighted “paracetamol,” “dissolution,” and “pharmaceutical analysis” as the most prominent research themes, indicating that studies continue to prioritize drug release behavior, assay performance, and analytical method development. This emphasis is consistent with regulatory requirements for ensuring formulation consistency and bioavailability.

Notably, emerging research clusters such as “Systematic review,” “Meta‐analysis,” “Pharmacokinetics,” and “Drug delivery” show a sharp increase in publications between 2024 and 2025, with several categories demonstrating growth rates of 14%–42% during this period. This upwards trajectory suggests a shift toward evidence synthesis, advanced modeling approaches, and enhanced delivery technologies, reflecting a broader trend in pharmaceutical sciences toward integrated and data‐driven evaluation of drug performance. Despite this growing activity, the bibliometric map shows relatively limited representation of studies addressing heavy metal contamination, microbial limit testing, or compliance within accreditation frameworks such as MS ISO/IEC 17025. These gaps support the relevance of the present study, which integrates physicochemical testing, dissolution profiling, UHPLC quantification, elemental impurity analysis, microbiological evaluation, and MS ISO/IEC 17025‐accredited laboratory practices. Therefore, the bibliometric results not only provide a quantitative overview of research trends but also justify the need for broader, safety‐oriented, and accreditation‐based approaches in future pharmaceutical quality‐control studies. These gaps are noteworthy given the regulatory importance of these parameters and highlight the need for more research in areas that directly support public health protection and analytical standardization. Furthermore, the scarcity of studies linking quality control outcomes with internationally accredited laboratory practices suggests an opportunity for future research to bridge methodological variability across regions. Strengthening research output in accreditation‐related testing would help harmonize analytical standards, improve data comparability, and support regulatory decision‐making, particularly in low‐ and middle‐income settings where substandard medicines remain a significant concern.

**Figure 4:**
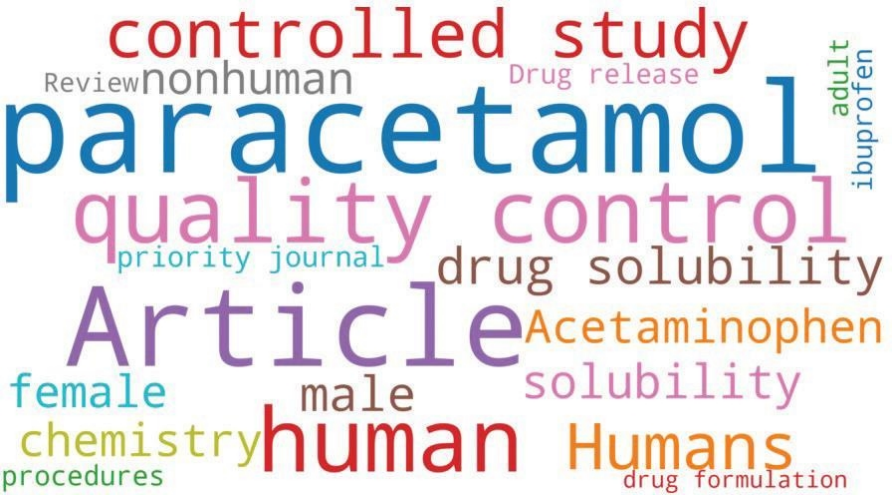
Word cloud analysis.

The keyword analysis (Figure 4) highlights “paracetamol,” “quality control,” and “article” as dominant terms, reflecting the strong research focus on analytical evaluation, regulatory compliance, and publication trends within pharmaceutical science. The frequent appearance of “drug solubility,” “solubility,” and “drug formulation” indicates that dissolution behavior and formulation performance remain central themes in paracetamol research, which is consistent with its importance in ensuring therapeutic efficacy and bioavailability. The presence of population‐related terms such as “human,” “Humans,” “male,” “female,” and “adult” suggests that a substantial proportion of studies link analytical findings to clinical or biopharmaceutical implications, whereas “controlled studies” emphasize structured experimental designs. Keywords such as “chemistry,” “procedures,” and “drug release” further reflect interest in chemical characterization, analytical methodologies, and performance testing. The appearance of related compounds such as “ibuprofen” and alternate drug names such as “acetaminophen” indicates comparative evaluations and synonym usage within the literature. Collectively, the keyword cloud demonstrates that global research on paracetamol is strongly oriented toward quality control, formulation science, and clinical relevance but also reveals limited emphasis on safety‐related domains such as heavy metals and microbial testing, highlighting an existing gap that aligns with the broader findings of this bibliometric study. Overall, the bibliometric findings underscore both the progress achieved and the current underexplored domains in paracetamol quality control, positioning this study as a valuable contribution to filling these gaps by integrating accredited analytical testing with global research mapping.

## 4. Conclusion

The physicochemical evaluation of Dyna paracetamol tablets confirmed that the product met all the requirements of the BP and DRGD specifications. These findings support the consistent quality and efficacy of the tablets, underscoring the importance of routine quality control in pharmaceutical manufacturing. This study confirms the quality of Dyna, Homecare Poro, and Paracil paracetamol tablets on the basis of BP through validated analytical testing performed under MS ISO/IEC 17025 conditions. All the parameters tested were within the pharmacopeial limits, indicating good formulation quality and proper manufacturing practices. The use of an accredited laboratory framework adds robustness to the findings, enhancing the credibility of the data for regulatory, industrial, and clinical use.

This study provides a comprehensive evaluation of Dyna, Homecare Poro, and Paracil paracetamol tablets through MS ISO/IEC 17025‐accredited physicochemical, heavy metal, and microbiological analyses, which demonstrated full compliance with the BP and DRGD standards. All measured parameters, including tablet weight, hardness, friability, disintegration time, heavy metal content, and microbial counts, were within acceptable limits, confirming the overall safety and quality of the tested formulation.

The inclusion of dissolution testing and UHPLC analysis strengthened the chemical evaluation of the tested products. Dissolution testing using the BP paddle method showed variable release patterns among the three brands, with Dyna achieving the highest final drug release of 100% at 60 min, whereas Homecare Poro and Paracil showed lower release values at the same time point. However, the noncumulative release trends observed, particularly for Homecare Poro and Paracil, indicate that the dissolution data should be interpreted cautiously and that further method refinement, replicate testing, blank correction, and validated chromatographic confirmation of dissolution samples are recommended. UHPLC‐DAD analysis at 275 nm successfully detected paracetamol in all three brands, with distinct chromatographic peaks and excellent calibration linearity. Dyna showed the highest peak area response and estimated concentration, followed by Homecare Poro and Paracil, supporting the presence and quantifiable detection of the active pharmaceutical ingredient in the tested tablets.

The analyses in this study were conducted under MS ISO/IEC 17025‐accredited laboratory conditions, which provides assurance that the testing procedures followed established quality management and technical competency requirements. However, ISO/IEC 17025 accreditation reflects the competence of a laboratory to perform specific tests within its accredited scope and does not, by itself, replace the value of comparative testing across multiple laboratories. Interlaboratory comparison is important because it allows assessment of reproducibility, method robustness, and consistency of results when the same product is analyzed under similar standard procedures by different accredited laboratories. Therefore, although the present findings support the compliance of the tested paracetamol tablets with relevant quality requirements, the inclusion of interlaboratory data would provide stronger evidence of analytical reliability and broader confidence in the reported results. A limitation of the present study is that the physicochemical, dissolution, chromatographic, heavy metal, and microbiological evaluations were performed within a single laboratory setting. Although the analyses were carried out under MS ISO/IEC 17025‐accredited laboratory conditions, no interlaboratory comparison was conducted. As a result, the reproducibility of the findings across different accredited laboratories could not be assessed. Future studies should include comparative testing involving two or more MS ISO/IEC 17025‐accredited laboratories to evaluate interlaboratory variability, confirm method robustness, and strengthen the reliability of pharmaceutical quality assessment data.

Complementing these laboratory findings, the bibliometric analysis of 2191 Scopus‐indexed publications (2010–2025) reveals strong global research activity centered on dissolution testing, pharmaceutical analysis, and evidence synthesis, alongside emerging interest in pharmacokinetics, drug delivery, and systematic reviews. However, the bibliometric dataset also highlights notable gaps, particularly in research related to heavy metal evaluation, microbiological quality, and the use of accredited laboratories in pharmaceutical quality assessment. These underrepresented areas underscore the importance of integrating robust analytical frameworks such as MS ISO/IEC 17025 accreditation into future studies to enhance methodological reliability, regulatory relevance, and global harmonization. Collectively, the combined analytical‐bibliometric approach used in this study strengthens the evidence base for paracetamol quality control and provides direction for future research aimed at safeguarding medicine quality, supporting regulatory oversight, and improving public health outcomes. Overall, the integrated analytical bibliometric approach used in this study supports the quality and safety assessment of Dyna, Homecare Poro, and Paracil paracetamol tablets and provides direction for future studies involving larger sample sizes, interlaboratory comparison, validated dissolution methods, and broader accredited testing frameworks to strengthen regulatory confidence and public health protection.

## Funding

No funding was received for this manuscript.

## Ethics Statement

Ethics approval was not required for this study because it involved only laboratory testing of commercially available paracetamol tablets and analysis of publicly accessible bibliometric data.

## Conflicts of Interest

The authors declare no conflicts of interest.

## Data Availability

The data that support the findings of this study are available on request from the corresponding author. The data are not publicly available due to privacy or ethical restrictions.

## References

[1] Krenzelok E. P. , Preventing Inpatient Acetaminophen Overexposure, American Journal of Health-System Pharmacy. (2016) 73, no. 15, 1123–1124, 10.2146/ajhp160328. 27267536

[2] Dandekar M. and Dandekar P. , Ensuring Excellence: Quality Assurance in the Pharmaceutical Industry, International Journal of Pharmaceutical Quality Assurance. (2024) 15, no. 4, 10.25258/ijpqa.15.4.24.

[3] Masheta D. Q. , Alazzawi S. K. , and Bash A. A. , Comparative Quality Control Study of Widely Used Brands of Paracetamol Tablets in Iraq, Maaen Journal for Medical Sciences. (2022) 1, no. 1, 10.55810/2789-9128.1009.

[4] Khawory M. H. , Subki M. F. M. , Shahudin M. A. , Sofian N. H. A. S. , Latif N. H. , Salin N. H. , Zobir S. Z. M. , and Noordin M. I. , Physico-Chemicals Characterization of Quercetin From the Carica papaya Leaves by Different Extraction Techniques, Open Journal of Physical Chemistry. (2021) 11, no. 3, 129–143, 10.4236/ojpc.2021.113007.

[5] Adepoju-Bello A. A. , Olaofe Oyawaluja B. , and Chiwendu Ndugba A. , Physical Assessment and Atomic Absorption Spectrophotometric Analysis of Cadmium and Lead In Twenty-Five Brands of Ciprofloxacin Tablets Marketed In Lagos, Nigeria, African Journal of Pharmaceutical Research and Development. (2024) 16, no. 1, 100–108, 10.59493/ajopred/2024.1.11.

[6] Polarine J. and McCall J. , Microbiological Concerns in Non-Sterile Manufacturing, Disinfection and Decontamination: A Practical Handbook, 2018, CRC Press, 135–147, 10.1201/9781351217026-8.

[7] Salih Q. M. , Developing an Analytical Method Using Atomic Absorption Spectroscopy to Estimate Some Heavy Metals in Medicines Nutritional Supplements and Blood, International Journal of Advanced Chemistry Research. (2024) 6, no. 1, 95–101, 10.33545/26646781.2024.v6.i1b.172.

[8] Nagpure N. , Raut H. , Askar D. , Narange N. , Bhurse S. , and Kasliwal D. , Navigating The N-Nitrosamine Impurities Challenge: Comprehensive Strategies For Ensuring Paracetamol Purity Through Analytical Advances, Control Measures, And Emerging Mitigation Trends, Educational Administration: Theory and Practice. (2024) 30, no. 4, 1003–1011, 10.53555/kuey.v30i4.10763.

[9] Medicines and Healthcare Products Regulatory Agency (MHRA) , The British Pharmacopoeia 2022 (Volume V: Infrared Reference Spectra, Appendices, Supplementary Chapters, Index), 2022, The Stationery Office.

[10] Ruiz-Rosero J. , Ramirez-Gonzalez G. , and Viveros-Delgado J. , Software survey: ScientoPy, a Scientometric Tool for Topics Trend Analysis in Scientific Publications, Scientometrics. (2019) 121, no. 2, 1165–1188, 10.1007/s11192-019-03213-w.

[11] Kumar Manglavat S. , Kumawat D. , and Goswami R. , Process Validation for Antiviral Famciclovir Tablet, Asian Journal of Pharmacy and Technology. (2020) 10, no. 3, 170–174, 10.5958/2231-5713.2020.00029.X.

[12] Read E. K. , Shah R. B. , Riley B. S. , Park J. T. , Brorson K. A. , and Rathore A. S. , Process Analytical Technology (PAT) for Biopharmaceutical Products: Part II. Concepts and Applications, Biotechnology and Bioengineering. (2010) 105, no. 2, 285–295, 10.1002/bit.22529, 19731253. 19731253

[13] Tidswell E. C. , Tirumalai R. S. , and Gross D. D. , Clarifications on the Intended Use of USP <61> Microbiological Examination of Nonsterile Products: Microbial Enumeration Tests, PDA Journal of Pharmaceutical Science and Technology. (2024) 78, no. 3, 348–357, 10.5731/PDAJPST.2023.012855, 38942475. 38942475

